# Sodium-glucose cotransporter-2 inhibitors use and the risk of gout: a systematic review and meta-analysis

**DOI:** 10.3389/fendo.2023.1158153

**Published:** 2023-05-23

**Authors:** Shih-Wei Lai, Bing-Fang Hwang, Yu-Hung Kuo, Chiu-Shong Liu, Kuan-Fu Liao

**Affiliations:** ^1^ Department of Public Health, College of Public Health, China Medical University, Taichung, Taiwan; ^2^ Department of Medicine, College of Medicine, China Medical University, Taichung, Taiwan; ^3^ Department of Family Medicine, China Medical University Hospital, Taichung, Taiwan; ^4^ Department of Occupational Safety and Health, College of Public Health, China Medical University, Taichung, Taiwan; ^5^ Department of Research, Taichung Tzu Chi Hospital, Taichung, Taiwan; ^6^ College of Medicine, Tzu Chi University, Hualien, Taiwan; ^7^ Division of Hepatogastroenterology, Department of Internal Medicine, Taichung Tzu Chi Hospital, Taichung, Taiwan

**Keywords:** diabetes mellitus, gout, meta-analysis, sodium-glucose cotransporter-2 inhibitor (SGLT2i), randomized controlled trials (RCT)

## Abstract

**Objective:**

To assess the relationship between use of sodium-glucose cotransporter-2 inhibitors (SGLT2i) and the risk of gout among patients with type 2 diabetes mellitus (T2DM).

**Methods:**

A systemic review and meta-analysis were designed by reviewing articles published between 2000 January 1 and 2022 December 31 using PubMed system and Web of Science system based on the PRISMA 2020 guidelines. The end point of interest was gout (including gout flares, gout events, starting uric-acid lowering therapy and starting anti-gout drugs use) among patients with T2DM using SGLT2i versus not using SGLT2i. A random-effects model was utilized to measure the pooled hazard ratio (HR) with 95% confidence interval (CI) for the risk of gout associated with SGLT2i use.

**Results:**

Two prospective post-hoc analyses of randomized controlled trials and 5 retrospective electronic medical record-linkage cohort studies met the inclusion criteria. The meta-analysis demonstrated that there was a decreased risk of developing gout for SGLT2i use as comparing with non-use of SGLT2i among patients with T2DM (pooled HR=0.66 and 95%CI=0.57-0.76).

**Conclusions:**

This meta-analysis demonstrates that SGLT2i use is associated with a 34% decreased risk of developing gout among patients with T2DM. SGLT2i may be the treatment options for patients with T2DM who are at high risk of gout. More randomized controlled trials and real-world data are needed to confirm whether there is a class effect of SGLT2i for the risk reduction of gout among patients with T2DM.

## Introduction

Sodium-glucose cotransporter-2 inhibitors (SGLT2i) belong to a new development of oral anti-diabetic drug to treat persons with type 2 diabetes mellitus (T2DM). SGLT2i also demonstrates other beneficial effects on cardiorenal protection including the risk reduction for atherosclerotic events, hospitalization and progression of heart failure, and progression of chronic kidney disease (1–4). In addition to these pleiotropic effects mentioned above, recent studies demonstrated that SGLT2i has a uric-acid lowering effect and then such an effect seems to be a class effect for SGLT2i (5–11).

Although the academic community has not yet established a consensus, it is generally agreed that when the serum uric acid value is greater than or equal to 6.8 mg/dL, it is called hyperuricemia (12, 13). Hyperuricemia is linked to the development of gout (14). Theoretically, SGLT2i use can decrease the risk of developing gout based on the uric-acid lowering effect. However, clinical data demonstrated conflicting results about the relation between SGLT2i use and the risk of gout. Some demonstrated benefit (15–20), but some demonstrated no benefit (21). For example, a prospective post-hoc analysis of randomized controlled trials by Li et al. demonstrated that canagliflozin use (one SGLT2i) correlated with a reduced risk of gout in persons with T2DM (hazard ratio=0.53, 95% confidence interval=0.40-0.71) (15). A retrospective electronic medical record-linkage cohort study by Subramanian et al. demonstrated that no risk difference for gout was noted among patients with T2DM receiving SGLT2i compared with those receiving dipeptidyl peptidate-4 inhibitors (hazard ratio=1.10, 95% confidence interval=0.71-1.68) (21).

These conflicting results raise concern and also inspire efforts to find solution. In view of the conflicting evidence, a systematic review and meta-analysis was designed to check the relationship between SGLT2i use and the risk of gout among patients with T2DM.

## Methods

### Search strategy

A systemic review and meta-analysis was designed by reviewing articles published between 2000 January 1 and 2022 December 31 using PubMed system and Web of Science system based on the PRISMA 2020 guidelines (22). The following keywords were selected to find articles of interest:”sodium-glucose cotransporter”, “sodium-glucose transport”, “canagliflozin”, “dapagliflozin”, “empagliflozin”, “ertugliflozin”, “ipragliflozin”, “luseogliflozin”, “tofogliflozin”, “gout” and “uric acid”. These keywords were used in combination as following strategies: sodium-glucose cotransporter [title] AND gout [title], sodium-glucose transport [title] AND gout [title], canagliflozin [title] AND gout [title], dapagliflozin [title] AND gout [title], empagliflozin [title] AND gout [title], ertugliflozin [title] AND gout [title], ipragliflozin [title] AND gout [title], luseogliflozin [title] AND gout [title], tofogliflozin [title] AND gout [title], sodium-glucose cotransporter [title] AND uric acid [title], sodium-glucose transport [title] AND uric acid [title], canagliflozin [title] AND uric acid [title], dapagliflozin [title] AND uric acid [title], empagliflozin [title] AND uric acid [title], ertugliflozin [title] AND uric acid [title], ipragliflozin [title] AND uric acid [title], luseogliflozin [title] AND uric acid [title], as well as tofogliflozin [title] AND uric acid [title].

### Inclusion and exclusion criteria

The following inclusion criteria were addressed to find articles of interest for meta-analysis: (1) randomized controlled trials (RCTs) and/or post-hoc analysis of RCTs which selected subjects with T2DM investigating individual SGLT2i and/or all SGLT2i; (2) observational studies (including cohort and case-control studies) which selected subjects with T2DM investigating individual SGLT2i and/or all SGLT2i; (3) the end point of interest was gout (including gout flares, gout events, starting uric-acid lowering therapy and starting anti-gout drugs use) among subjects with T2DM using SGLT2i versus not using SGLT2i; (4) the hazard ratio or odds ratio of gout was shown.

**Figure 1:**
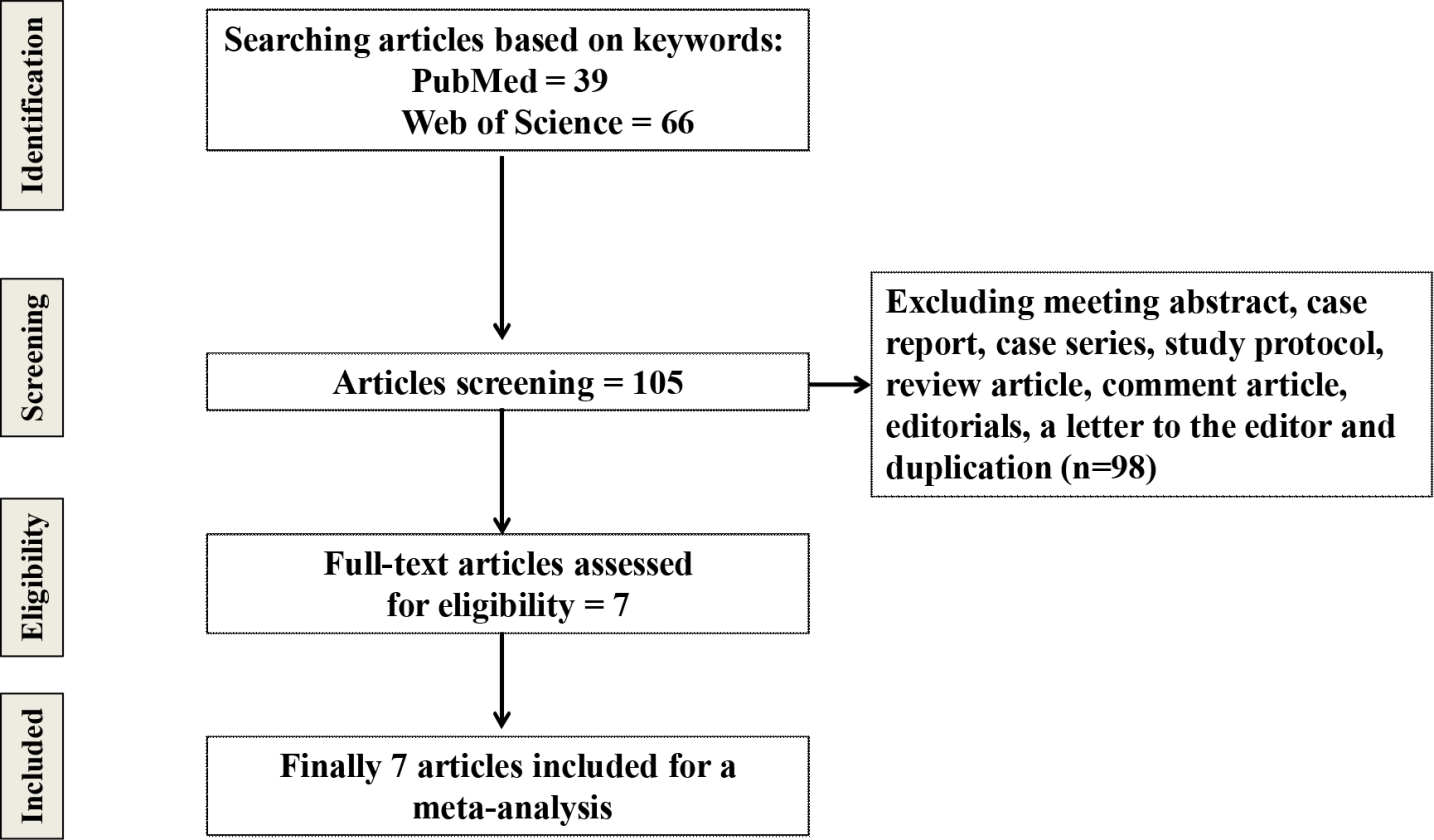
Flow chart of searching articles.

The exclusion criteria were applied as follows: (1) meeting abstract, case report, case series, study protocol, review article, comment article, editorials and a letter to the editor; (2) data were not fully demonstrated; (3) research without peer review (**Figure 1**).

### Data extraction

Two authors (KFL and YHK) assessed the eligibility of all found articles according to the above inclusion and exclusion criteria. The following terms were extracted: the surname of first author, study population, baseline characteristics of study subjects (including mean age and male percentage), number of SGLT2i use, number of comparator use, treatment/follow-up duration, and adjusted hazard ratio (HR) with 95% confidence interval (CI). Two authors (BFH and CSL) discussed together to resolve the conflicting opinions.

### Assessment of research quality

The Newcastle-Ottawa Scale system was utilized to check the quality and the risk of bias of the observational studies included (23). The Cochrane Collaboration’s tool was utilized to check the quality and the risk of bias of RCTs and/or post-hoc analysis of RCTs (24).

### Statistical analysis

A random-effects model was done to estimate the pooled HR with 95%CI for the risk of gout associated with SGLT2i use versus non-use of SGLT2i. Three sub-analyses were performed to measure the subtotal HR with 95%CI for the risk of gout associated with SGLT2i use versus placebo, SGLT2i use versus glucagon-like peptide-1 receptor agonists (GLP1RA), and SGLT2i use versus dipeptidyl peptidate-4 inhibitors (DPP4i), respectively. The I^2^ statistics were used to check the heterogeneity between included studies. The I^2^ value > 50% indicates that there could be a significant heterogeneity between included studies (25). The statistical analyses were performed by the aid of RStudio and the meta package (26, 27). The P value < 0.05 indicates statistically significant.

## Results

### Characteristics of included studies

**Table 1** lists the characteristic information of the 7 eligible studies. There were 2 prospective post-hoc analyses of RCTs and 5 retrospective electronic medical record-linkage cohort studies.

**Table 1:** Characteristics of included studies in a met-analysis.

First author (year)	Population	Baseline characteristics (SGLT2i vs comparator)	SGLT2i (n)	Comparator (n)	Treatment/ follow-up duration	Adjusted HR (95% CI) of gout
Prospective post-hoc analysis of randomized controlled trials (SGLT2i vs placebo)						
Li et al. (2019) (19)	T2DM	Age(63.2 ± 8.3 vs 63.4 ± 8.2 years), male(65% vs 63%)	Canagliflozin 100 or 300 mg daily(n=5795)	Placebo (n=4347)	3.6 years	0.53 (0.40-0.71)
Ferreira et al. (2022) (16)	T2DM	Age(63.1 ± 8.6 vs 63.2 ± 8.8 years), male(71.2% vs 72%)	Empagliflozin 10 or 25 mg daily(n=4687)	Placebo (n=2333)	Median 2.6 years	0.67 (0.53-0.85)
Retrospective electronic medical record-linkage cohort studies (SGLT2i vs GLP1RA)						
Fralick et al. (2020) (17)	T2DM	Age(54.22 ± 9.85 vs 54.23 ± 10.08 years), male(48% vs 48.3%)	Any SGLT2i (n=119530)	Any GLP1RA (n=119530)	9 months	0.64 (0.57-0.72)
Lund et al. (2021) (18)	T2DM	Median age(59 vs 59 years), male(57% vs 58%)	Any SGLT2i (n=11047)	Any GLP1RA (n=11047)	3 years	0.58 (0.44-0.75)
Retrospective electronic medical record-linkage cohort studies (SGLT2i vs DPP4i)						
Fralick et al. (2020) (17)	T2DM	Not available	Any SGLT2i (n=97442)	Any DPP4i (n=97442)	9 months	0.66 (0.58-0.75)
Lund et al. (2021) (18)	T2DM	Median age(61 vs 60 years), male(62% vs 62%)	Any SGLT2i (n=9694)	Any DPP4i (n=9694)	15010 vs 15076 person-years	0.60 (0.44-0.82)
Chung et al. (2021) (19)	T2DM	Age (57.72±12.15 vs 57.75±12.08 years), male(53.72% vs 53.64%)	Any SGLT2i n=47405)	Any DPP4i (n=47405)	2.5 years	0.89 (0.82-0.96)
Zhou et al. (2023) (20)	T2DM	Age(58.2±10.9 vs 60.0±10.9 years), male(57.57% vs 51.26%)	Any SGLT2i (n=16144)	Any DPP4i (n=16144)	2.5 years	0.49 (0.42-0.58)
Subramanian et al. (2023) (21)	T2DM	Age(58.97±10.65 vs 58.97±11.56 years), male(57.13% vs 57.17%)	Any SGLT2i (n=8650)	Any DPP4i (n=8650)	15836 vs 14553 person-years	1.10 (0.71-1.68)

RCT, randomized controlled trial.

T2DM, type 2 diabetes mellitus.

HR, hazard ratio; 95%CI: 95% confidence interval.

SGLT2i, sodium-glucose cotransporter-2 inhibitors; GLP1RA, glucagon-like peptide-1 receptor agonists;

DPP4i, dipeptidyl peptidate-4 inhibitors.

Gout: including gout flares, gout events, starting uric-acid lowering therapy and starting anti-gout drugs use.

The 2 prospective post-hoc analyses of RCTs by Li et al. and by Ferreira et al. demonstrated a lower HR for gout associated with SGLT2i use as comparing with placebo, with reaching statistical significance (HR=0.53 and HR=0.67, respectively) (15, 16). The 4 retrospective electronic medical record-linkage cohort studies demonstrated a lower HR for gout associated with SGLT2i use as comparing with GLP1RA use or DPP4i use, with reaching statistical significance (17–20). But one retrospective electronic medical record-linkage cohort study by Subramanian et al. demonstrated an elevated HR for gout associated with SGLT2i use as comparing with DPP4i use, but not achieving statistical significance (HR=1.10 and 95%CI=0.71-1.68) (21).

The 2 prospective post-hoc analyses of RCTs had a low risk of bias from the Cochrane Collaboration’s tool. The 5 retrospective electronic medical record-linkage cohort studies had high-quality with a low risk of bias based on the Newcastle-Ottawa Scale system.

### Pooled hazard ratio of gout

**Figure 2:**
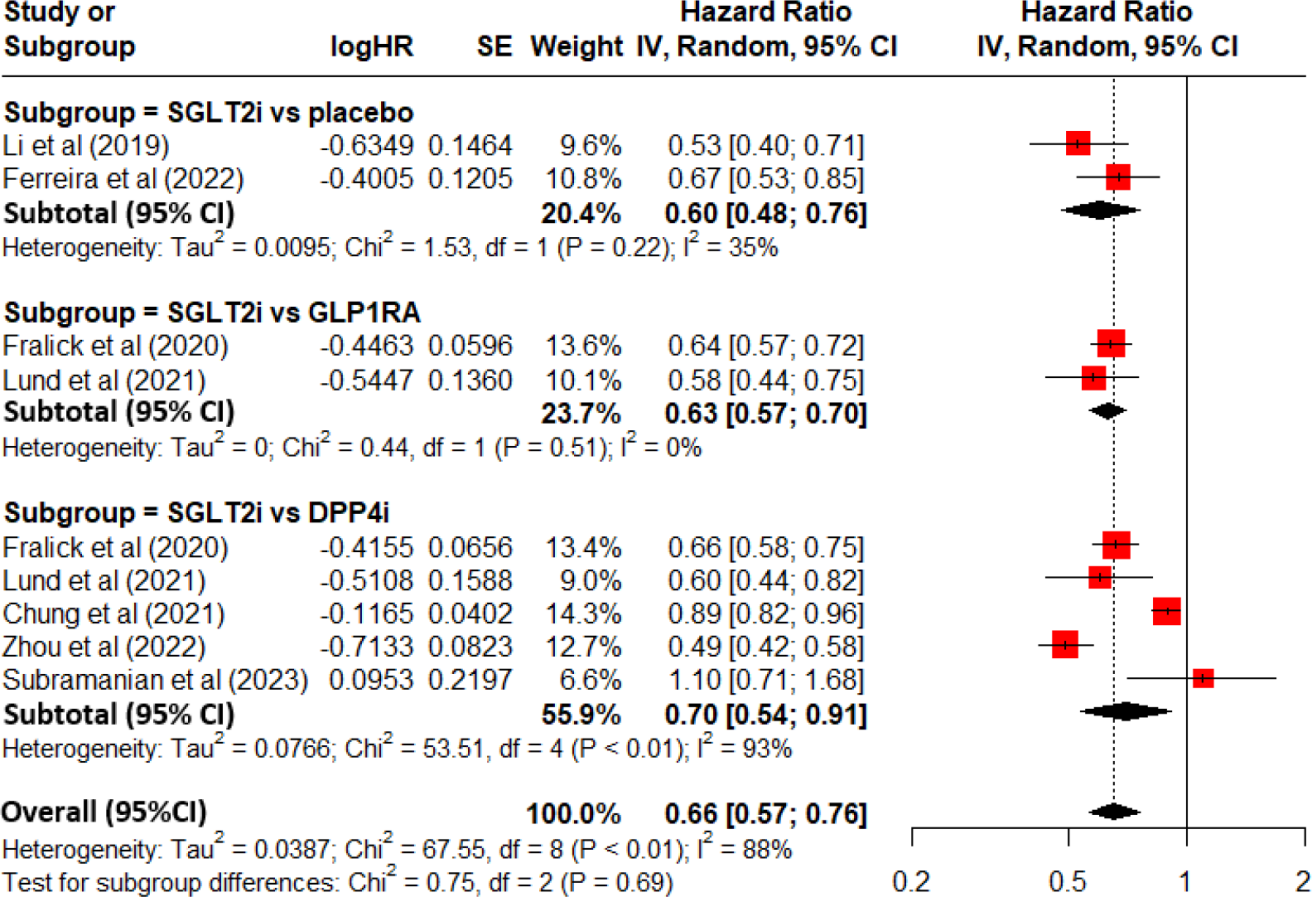
Forest plot for total and subgroup analyses revealing the effect of SGLT2i on the development of gout among patients with T2DM as comparing with placebo or other anti-diabetic drugs.

**Figure 2** demonstrates a forest plot with the pooled HR and 95%CI for the risk of gout. Overall, there was a decreased risk of developing gout for SGLT2i use as comparing with non-use of SGLT2i among patients with T2DM (pooled HR=0.66, 95%CI=0.57-0.76 and P<0.01). In sub-analysis for the 2 prospective post-hoc analyses of RCTs, there was a decreased risk of developing gout for SGLT2i use as comparing with placebo (subtotal HR=0.60 and 95%CI=0.48-0.76). In the sub-analysis for the 2 retrospective electronic medical record-linkage cohort studies (SGLT2i vs. GLP1RA), there was a decreased risk of developing gout for SGLT2i use as comparing with GLP1RA use (subtotal HR=0.63 and 95%CI=0.57-0.70). In the sub-analysis for 5 retrospective electronic medical record-linkage cohort studies (SGLT2i vs. DPP4i), there was a decreased risk of developing gout for SGLT2i use as comparing with DPP4i use (subtotal HR=0.70 and 95%CI=0.54-0.91).

There was a significant heterogeneity between included studies (I2 = 88% and P<0.01).

### Assessment of publication bias

**Figure 3:**
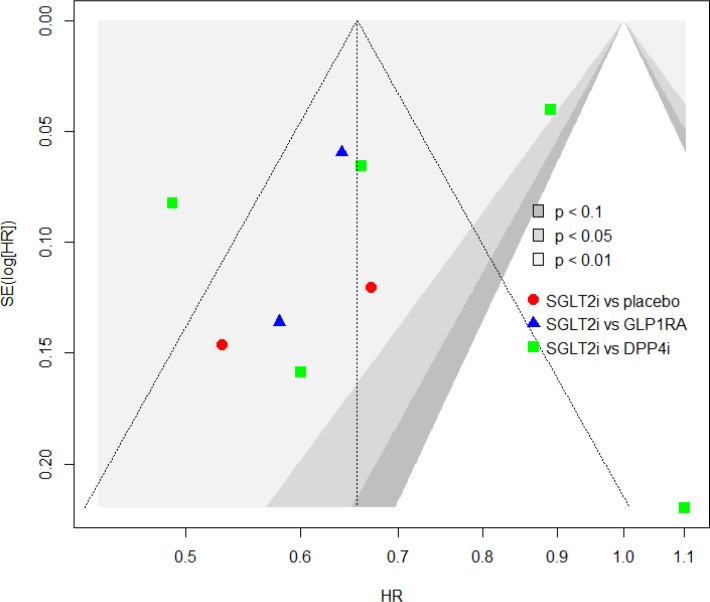
Funnel plot revealing the assessment of publication bias.

The funnel plot is presented in **Figure 3**. A visual inspection demonstrates symmetry. It indicates that there was no publication bias. These results were confirmed by The Begg’s test (P=0.8348) and the Egger’s test (P=0.1937) (28, 29).

### Sensitivity analysis

One study from Subramanian et al. demonstrated an elevated HR for gout associated with SGLT2i use as comparing with DPP4i use, but not achieving statistical significance (21). After excluding Subramanian et al’s study, a pooled HR was 0.63(95%CI =0.55-0.73 and P<0.001). It still achieved statistical significance.

## Discussion

This present meta-analysis demonstrated that there was a 34% decreased risk of developing gout for SGLT2i use as comparing with non-use of SGLT2i among patients with T2DM. In sub-analysis, no matter comparing with placebo, GLP1RA use or DPP4i use, the risk reduction of gout remained to be observed. Based on the above findings, SGLT2i may be the treatment options for patients with T2DM who are at high risk of gout, including those patients with a history of gout, high levels of serum uric acid and/or other risk factors for gout.

The potential mechanisms underlying the use of SGLT2i, and risk reduction of gout are not fully understood. We review the literature and summarize as below. It is well known that lowering serum uric acid levels is a key goal for the prevention and treatment of gout. SGLT2i works by blocking the reabsorption of glucose and sodium in the proximal tubule of the kidney, leading to increased urinary glucose and sodium excretion, and increased urinary volume, which then lowers blood glucose levels. This increased urinary volume may also enhance uric acid excretion, which could explain the reduction in blood uric acid levels (30–32). It is a rational hypothesis that SGLT2i reduces blood uric acid levels by enhancing urinary excretion of uric acid. Thus, the risk of gout is reduced after the use of SGLT2i.

Some caveats and limitations should be discussed. First, recently many real-world studies utilized the electronic medical record-linkage database for analysis. The immortal time bias is possibly encountered when using such a database (33). If the immortal time bias is not managed correctly during the stages of research design and research analysis, the outcome results can lead to the wrong direction (34–36). Some research included the immortal time into the treatment group, but some research simply excluded the immortal time from the research (34–36). All of these methods may overestimate of the benefit of the studied drug (34–36). How the immortal time bias was handled was not mentioned in the method section of the 5 retrospective electronic medical record-linkage cohort studies. Therefore, interpretation of their results should be cautious. The RCTs can avoid the immortal time bias. In order to overcome the potential immortal time bias which could be present in the 5 retrospective electronic medical record-linkage cohort studies, the sub-analysis of the 2 prospective post-hoc analyses of RCTs demonstrated that there was a risk reduction of gout associated with SGLT2i use. These findings partially support the results of the 5 retrospective electronic medical record-linkage cohort studies that SGLT2i use could be associated with a risk reduction of gout when comparing with GLP1RA use or DPP4i use. Second, there are 7 SGLT2i available in the markets, including canagliflozin, dapagliflozin, empagliflozin, ertugliflozin, ipragliflozin, luseogliflozin and tofogliflozin. Six of these 7 drugs show a uric acid-lowering effect (5–11). It seems to be a class effect of SGLT2i for lowering uric acid. Currently, only two individual SGLT2i (canagliflozin and empagliflozin) had performed the post-hoc analyses of RCTs on the risk of gout. The other 5 SGLT2i did not demonstrate the post-hoc analyses of RCTs on the risk of gout. Further RCTs or post-hoc analyses of RCTs are needed to clarify whether there is a class effect of SGLT2i for the risk reduction of gout. Third, the heterogeneity of the 2 prospective post-hoc analyses of RCTs and the 2 retrospective electronic medical record-linkage cohort studies (SGLT2i vs. GLP1RA) was low, but the heterogeneity of all included studies seemed to be high in this meta-analysis. Some potential sources of heterogeneity should be mentioned. For example, differences in study populations, differences in study design, differences in intervention characteristics, or differences in outcome measures, might contribute to heterogeneity across studies (37, 38).

## Conclusion

This present meta-analysis demonstrates that SGLT2i use is associated with a 34% decreased risk of developing gout among patients with T2DM. SGLT2i may be the treatment options for patients with T2DM who are at high risk of gout. More RCTs and real-world data are needed to confirm whether there is a class effect of SGLT2i for the risk reduction of gout among patients with T2DM.

## Data availability statement

The original contributions presented in the study are included in the article/supplementary material. Further inquiries can be directed to the corresponding author.

## Author contributions

S-WL contributed to the conception of the study, initiated the draft of the study, and approved the final draft. Y-HK and K-FL conducted data analysis. B-FH and C-SL interpreted the data. All authors contributed to the article and approved the submitted version.

## References

[1] Verma S McMurray JJV . SGLT2 inhibitors and mechanisms of cardiovascular benefit: a state-of-the-art review. Diabetologia. (2018) 61:2108–17. doi: 10.1007/s00125-018-4670-7 30132036

[2] Heerspink HJL Kosiborod M Inzucchi SE Cherney DZI . Renoprotective effects of sodium-glucose cotransporter-2 inhibitors. Kidney Int (2018) 94:26–39. doi: 10.1016/j.kint.2017.12.027 29735306

[3] Zelniker TA Braunwald E . Mechanisms of cardiorenal effects of sodium-glucose cotransporter 2 inhibitors: JACC state-of-the-Art review. J Am Coll Cardiol (2020) 75:422–34. doi: 10.1016/j.jacc.2019.11.031 32000955

[4] Zelniker TA Braunwald E . Clinical benefit of cardiorenal effects of sodium-glucose cotransporter 2 inhibitors: JACC state-of-the-Art review. J Am Coll Cardiol (2020) 75:435–47. doi: 10.1016/j.jacc.2019.11.036 32000956

[5] Davies MJ Trujillo A Vijapurkar U Damaraju CV Meininger G . Effect of canagliflozin on serum uric acid in patients with type 2 diabetes mellitus. Diabetes Obes Metab (2015) 17:426–9. doi: 10.1111/dom.12439 PMC5054919 25600248

[6] McDowell K Welsh P Docherty KF Morrow DA Jhund PS de Boer RA . Dapagliflozin reduces uric acid concentration, an independent predictor of adverse outcomes in DAPA-HF. Eur J Heart Fail (2022) 24:1066–76. doi: 10.1002/ejhf.2433 PMC9540869 35064721

[7] Doehner W Anker SD Butler J Zannad F Filippatos G Ferreira JP . Uric acid and sodium-glucose cotransporter-2 inhibition with empagliflozin in heart failure with reduced ejection fraction: the EMPEROR-reduced trial. Eur Heart J (2022) 43:3435–46. doi: 10.1093/eurheartj/ehac320 PMC9492270 35788657

[8] Tanaka M Yamakage H Inoue T Odori S Kusakabe T Shimatsu A . Beneficial effects of ipragliflozin on the renal function and serum uric acid levels in Japanese patients with type 2 diabetes: a randomized, 12-week, open-label, active-controlled trial. Intern Med (2020) 59:601–9. doi: 10.2169/internalmedicine.3473-19 PMC7086326 32115517

[9] Chino Y Kuwabara M Hisatome I . Factors influencing change in serum uric acid after administration of the sodium-glucose cotransporter 2 inhibitor luseogliflozin in patients with type 2 diabetes mellitus. J Clin Pharmacol (2022) 62:366–75. doi: 10.1002/jcph.1970 PMC9299189 34545949

[10] Ouchi M Oba K Kaku K Suganami H Yoshida A Fukunaka Y . Uric acid lowering in relation to HbA1c reductions with the SGLT2 inhibitor tofogliflozin. Diabetes Obes Metab (2018) 20:1061–5. doi: 10.1111/dom.13170 PMC5887894 29171930

[11] Akbari A Rafiee M Sathyapalan T Sahebkar A . Impacts of Sodium/Glucose cotransporter-2 inhibitors on circulating uric acid concentrations: a systematic review and meta-analysis. J Diabetes Res (2022) 2022:7520632. doi: 10.1155/2022/7520632 35224108 PMC8872662

[12] Hyndman D Liu S Miner JN . Urate handling in the human body. Curr Rheumatol Rep (2016) 18:34. doi: 10.1007/s11926-016-0587-7 27105641 PMC4841844

[13] Cutolo M Cimmino MA Perez-Ruiz F . Potency on lowering serum uric acid in gout patients: a pooled analysis of registrative studies comparing febuxostat vs. allopurinol. Eur Rev Med Pharmacol Sci (2017) 21:4186–95. 29028079

[14] Dalbeth N Choi HK Joosten LAB Khanna PP Matsuo H Perez-Ruiz F . Gout. Nat Rev Dis Primers. (2019) 5:69. doi: 10.1038/s41572-019-0115-y 31558729

[15] Li J Badve SV Zhou Z Rodgers A Day R Oh R . The effects of canagliflozin on gout in type 2 diabetes: a post-hoc analysis of the CANVAS program. Lancet Rheumatol (2019) 1:e220–e8. doi: 10.1016/S2665-9913(19)30078-5 38229378

[16] Ferreira JP Inzucchi SE Mattheus M Meinicke T Steubl D Wanner C . Empagliflozin and uric acid metabolism in diabetes: a *post hoc* analysis of the EMPA-REG OUTCOME trial. Diabetes Obes Metab (2022) 24:135–41. doi: 10.1111/dom.14559 PMC9293326 34558768

[17] Fralick M Chen SK Patorno E Kim SC . Assessing the risk for gout with sodium-glucose cotransporter-2 inhibitors in patients with type 2 diabetes: a population-based cohort study. Ann Intern Med (2020) 172:186–94. doi: 10.7326/M19-2610 PMC7217750 31931526

[18] Lund LC Højlund M Henriksen DP Hallas J Kristensen KB . Sodium-glucose cotransporter-2 inhibitors and the risk of gout: a Danish population based cohort study and symmetry analysis. Pharmacoepidemiol Drug Saf. (2021) 30:1391–5. doi: 10.1002/pds.5252 33881179

[19] Chung MC Hung PH Hsiao PJ Wu LY Chang CH Wu MJ . Association of sodium-glucose transport protein 2 inhibitor use for type 2 diabetes and incidence of gout in Taiwan. JAMA Netw Open (2021) 4:e2135353. doi: 10.1001/jamanetworkopen.2021.35353 34797368 PMC8605485

[20] Zhou J Liu X Chou OH Li L Lee S Wong WT . Lower risk of gout in sodium glucose cotransporter 2 (SGLT2) inhibitors versus dipeptidyl peptidase-4 (DPP4) inhibitors in type-2 diabetes. Rheumatol (Oxford) (2023) 62(4):1501–10. doi: 10.1093/eurheartj/ehac544.2681 36066415

[21] Subramanian A Gokhale K Sainsbury C Nirantharakumar K Toulis KA . Sodium-glucose cotransporter-2 inhibitors and the risk of gout in patients with type 2 diabetes mellitus: a propensity-score-matched, new-user design study with an active comparator using the IQVIA medical research data UK database. Diabetes Obes Metab (2023) 25:156–65. doi: 10.1111/dom.14858 PMC10087572 36056476

[22] Page MJ McKenzie JE Bossuyt PM Boutron I Hoffmann TC Mulrow CD . The PRISMA 2020 statement: an updated guideline for reporting systematic reviews. J Clin Epidemiol. (2021) 134:178–89. doi: 10.1016/j.jclinepi.2021.03.001 33789819

[23] Wells GA SB O'Connell D Peterson J Welch V Losos M . The Newcastle-Ottawa scale (NOS) for assessing the quality of nonrandomized studies in meta-analyses . Available at: http://www.ohri.ca/programs/clinical_epidemiology/oxford.asp.

[24] Higgins JP Sterne JA Savovic J Page MJ Hróbjartsson A Boutron I . A revised tool for assessing risk of bias in randomized trials. Cochrane Database systematic Rev (2016) 10:29–31.

[25] Higgins JP Thompson SG . Quantifying heterogeneity in a meta-analysis. Stat Med (2002) 21:1539–58. doi: 10.1002/sim.1186 12111919

[26] Posit-team. RStudio: integrated development environment for r. Posit Software,PBC,Boston,MA. Available at: http://www.posit.co.

[27] Balduzzi S Rücker G Schwarzer G . How to perform a meta-analysis with r: a practical tutorial. Evid Based Ment Health (2019) 22:153–60. doi: 10.1136/ebmental-2019-300117 PMC10231495 31563865

[28] Begg CB Mazumdar M . Operating characteristics of a rank correlation test for publication bias. Biometrics. (1994) 50:1088–101. doi: 10.2307/2533446 7786990

[29] Egger M Davey Smith G Schneider M Minder C . Bias in meta-analysis detected by a simple, graphical test. BMJ. (1997) 315:629–34. doi: 10.1136/bmj.315.7109.629 PMC2127453 9310563

[30] Scheen AJ . SGLT2 inhibitors: Benefit/Risk balance. Curr Diabetes Rep (2016) 16:92. doi: 10.1007/s11892-016-0789-4 27541294

[31] Bailey CJ . Uric acid and the cardio-renal effects of SGLT2 inhibitors. Diabetes Obes Metab (2019) 21:1291–8. doi: 10.1111/dom.13670 30762288

[32] Bonora BM Avogaro A Fadini GP . Extraglycemic effects of SGLT2 inhibitors: a review of the evidence. Diabetes Metab Syndr Obes (2020) 13:161–74. doi: 10.2147/DMSO.S233538 PMC6982447 32021362

[33] Bae JM . Statin intake and gastric cancer risk: an updated subgroup meta-analysis considering immortal time bias. J Prev Med Public Health (2022) 55:424–7. doi: 10.3961/jpmph.22.209 PMC9561136 36229904

[34] Suissa S . Immortal time bias in observational studies of drug effects. Pharmacoepidemiol Drug Saf. (2007) 16:241–9. doi: 10.1002/pds.1357 17252614

[35] Suissa S . Immortal time bias in pharmaco-epidemiology. Am J Epidemiol. (2008) 167:492–9. doi: 10.1093/aje/kwm324 18056625

[36] Targownik LE Suissa S . Understanding and avoiding immortal-time bias in gastrointestinal observational research. Am J Gastroenterol (2015) 110:1647–50. doi: 10.1038/ajg.2015.210 26323186

[37] Thompson SG Higgins JP . How should meta-regression analyses be undertaken and interpreted? Stat Med (2002) 21:1559–73. doi: 10.1002/sim.1187 12111920

[38] Ioannidis JP . Interpretation of tests of heterogeneity and bias in meta-analysis. J Eval Clin Pract (2008) 14:951–7. doi: 10.1111/j.1365-2753.2008.00986.x 19018930

